# Optical coherence tomography based microangiography as a non-invasive imaging modality for early detection of choroido-neovascular membrane in choroidal rupture

**DOI:** 10.1186/s40064-016-3161-x

**Published:** 2016-09-01

**Authors:** Kasra A. Rezaei, Qinqin Zhang, Jason Kam, Jin Liu, Ruikang K. Wang

**Affiliations:** 1 Department of Ophthalmology, University of Washington, 325 Ninth Avenue, Seattle, WA 98104 USA; 2 Department of Bioengineering, University of Washington, 3720 15th Ave NE, Seattle, WA 98195 USA

## Abstract

**Introduction:**

To evaluate and identify early microvascular changes in patient with choroidal rupture using optical coherence tomography (OCT) based microangiography (OMAG).

**Case description:**

One patient (one eye) with confirmed diagnosis of choroidal rupture after sustained ocular blunt trauma underwent OMAG imaging. OMAG was performed by Zeiss spectral domain OCT-angiography prototype using a “6.5 mm × 6.5 mm” field of view around macular region. The resulting images were presented into bilayers: the retinal layer and the choroidal layer.

**Discussion and evaluation:**

Choroidal rupture sites were easily shown on OMAG images with clear evidence of multiple breaks in Bruch’s membrane involving macula and the region superior to nerve. OMAG provided detailed vascular network patterns in the areas of choroidal rupture, showing a concern for choroidal neovascularization (CNV). The OMAG demonstrated cross sectional area to visualize CNV location relative to the other layers of the retina, identifying functional blood vessels through the lesion. The patient’s progress was followed using OMAG.

**Conclusion:**

The images provided by OMAG give detailed microvascular findings about the macula and adjacent retinal region along with the underlying choroidal alternations. In our case, details of the architecture and vascular flow of CNVM in choroidal rupture was delivered by OMAG, which were used to follow the progression of the disease progression. Further studies are needed to assess the role of quantitative and qualitative OCT microangiography in the evaluation and treatment of choroidal rupture.

## Background

Choroidal neovascularization (CNV) is a common complication associated with many disease states, including age-related macular degeneration, presumed ocular histoplasmosis syndrome (POHS), angioid streaks, pathologic myopia, posterior uveitis, and choroidal rupture. These new vessels lack endothelial tight junctions, which make them prone to leak, leading to possible exudation, hemorrhage, and serous retinal detachment. Ultimately, this would cause scarring and atrophy, giving irreversible visual morbidity.

Choroidal rupture may occur from direct contact at the site of impact, or indirect contact remotely from the site of impact (countercoup). Eighty percent of choroidal ruptures are indirect (Williams et al. 1990; Wood and Richardson 1990) that are generally associated with non-penetrating closed-globe blunt trauma. Due to its decreased elasticity relative to the retina and less tensile strength relative to the sclera, Bruch’s membrane is most susceptible to traumatic rupture. If Bruch’s membrane is damaged, patients are at risk for the development of CNV months or years after their initial injury. Up to 20 % of post traumatic patients can lead to CNV (Secretan et al. 1998).

Fluorescein angiography (FA) is an invasive imaging modality that has been used to assist physicians to identify CNV in patients with choroidal rupture. In our study we used a non-invasive imaging modality, optical coherence tomography based microangiography (OMAG) (Wang et al. 2010; An et al. 2010), to detect and follow the progression of the CNV.

## Methods

The patient underwent a complete eye examination including slit lamp examination, dilated fundus examination, color funding imaging, Heidelberg Spectralis OCT (Heidelberg Engineering GmbH, Heidelberg, Germany). Patient also underwent imaging with modified CIRRUS HD-5000 angiography prototype, containing a spectral domain OCT (SD OCTA) provided by Carl Zeiss Meditec Inc., (Dublin, CA, USA). The use of Cirrus HD-5000 angiography prototype in the imaging of the patients was approved by the University of Washington Institutional Review Board. The informed consent for the use of the unidentified personal and medical information for the publication of this case report and any accompanying images was obtained. All procedures adhered to the tenets of Declaration of Helsinki.

Cirrus HD-5000 SD-OCT system operates at a central wavelength of 840 nm and an A-scan speed of 68,000 scans/s. Three scanning protocols were performed, which included 512 × 128 macular raster scan, a single high density B-scan with 1024 A-scans and a montage OMAG scan. The montage scan was based on the tracking line scanning ophthalmoscope (LSO) built-in system and composed by 3 × 3 single cube scans. Each cube was of a field of view of 2.4 mm × 2.4 mm at the surface of retina. Transverse scanning was obtained with a total of 245 A-lines forming a single B scan. Four repeated B-scans were performed at each fixed location. Over a distance of 2.4 mm, 245 B scans were sampled with each scan measuring 9.8 μ apart. The acquisition rates were 222 frames/s (fps).

The adjacent cubes had 10 % overlap with each other that enabled the field of view of approx. 6.5 × 6.5 mm of 3 × 3 cubes after montaging. The OMAG algorithm (Huang et al. 2014) was applied to the volumetric dataset to obtain microstructural and microvascular images within retina and choroid. The average OCT B-scans were examined in the regular OCT data to show the retinal tissue in cross section as well as en-face projection images.

The retina and choroid were segmented into different distinct physiological layers (Yin et al. 2014). Three layers were segmented in retina, containing superficial retinal layer (GCL + IPL), middle retinal layer (INL + OPL) and deep retinal layer (ONL + ELM). The choroidal segment contains both the choriocapillaris and choroidal layers. The segmentation was performed using the OCT cross sectional structural images based on the intensity difference of the retinal layers. The three dimensional structure of the retina and microvasculature were rendered and projected using a 3D visualization software. To give better visualization, color coding was performed on different layer.

## Results

**Fig. 1:**
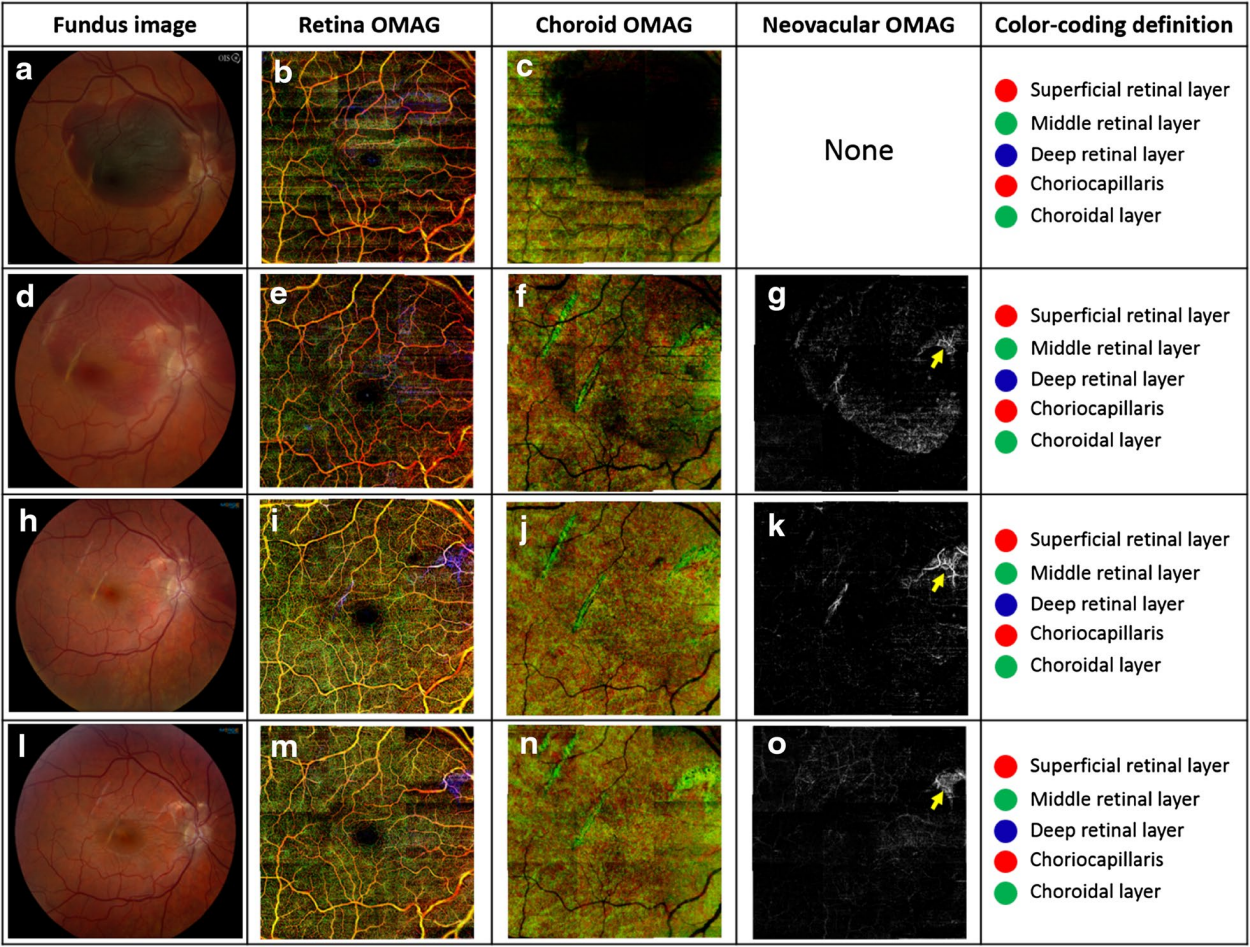
Color fundus photos of the right eye at presentation and OMAG image scan of the retina. **a**, **d**, **h**, **l** Show serial color fundus photos of the right eye, in progression postoperative month (POM) # 0, 1, 2 and 4, respectively. As the time elapses, there is visible clearing of the submacular hemorrhage s/p pneumatic displacement, however no obvious evidence of neovascular proliferation or gray subretinal tissue. **b**, **c**, **e**–**g**, **i**–**k**, **m**–**o** Show their respective OMAG image scans of approximately 6.5 × 6.5 mm^2^ in the macular region from the right eye. These images represented retinal microvasculature’s, presented in enface projection format, demonstrating the structural and microvascular information from the macula. Note that neovascular OMAG images were resulted from enface projecting the flow signals between RPE and Bruch’s membrane in which no blood vessels should be present in normal eyes

A 20-year-old Caucasian without prior ocular history presented with sustained blunt trauma to the right globe with a plastic wire with a metal tip. His best corrected visual acuity (BCVA) was count finger at 5″ in the affected right eye and 20/20 in the left eye. His intraocular pressures were 10 and 11 mmHg in the right and left eyes, respectively. His anterior slit lamp examination showed mild lid edema, clear cornea with 1+ AC cell in the right eye and left eye was unremarkable. On fundus examination, he was found to have a large area of sub-retinal blood, presented in the macula to the superior temporal arcade and crossing into the superior-nasal arcade adjacent to the nerve head (Fig. 1a). Left eye fundus exam was unremarkable (not shown).

Patient underwent pneumatic displacement of the sub-macular hemorrhage with C3F8 in office with face down positioning for 7 days. On the follow up examination three distinct areas of choroidal rupture were visualized and were evaluated using OMAG technology.

**Fig. 2:**
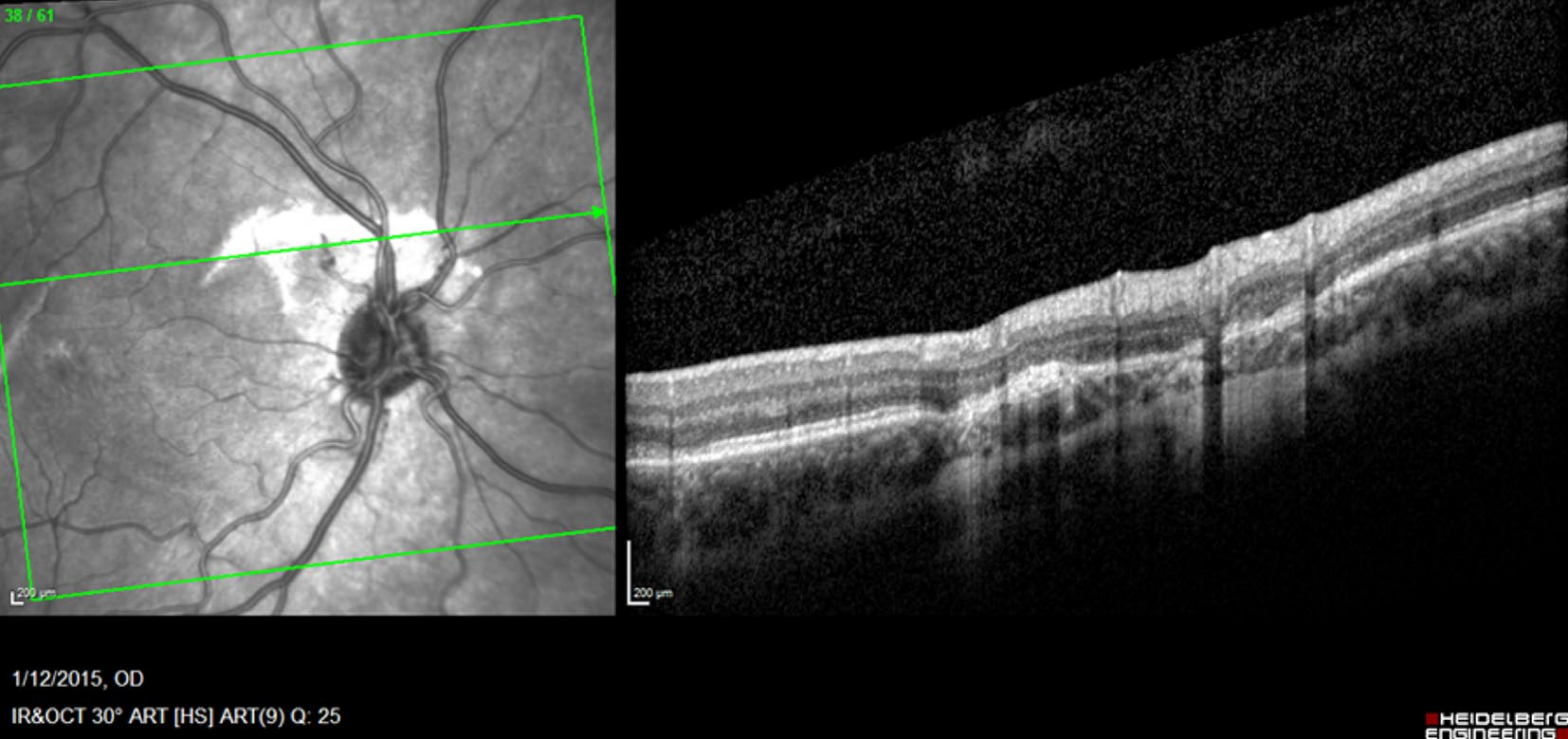
Heidelberg Spectralis OCT of POM 4 was done to evaluate the architecture of the retina and choroid and it shows the alteration of the retinal–choroidal structures in this case. The OCT image shows the disruption of the RPE layer with underlying Bruch’s membrane

**Fig. 3:**
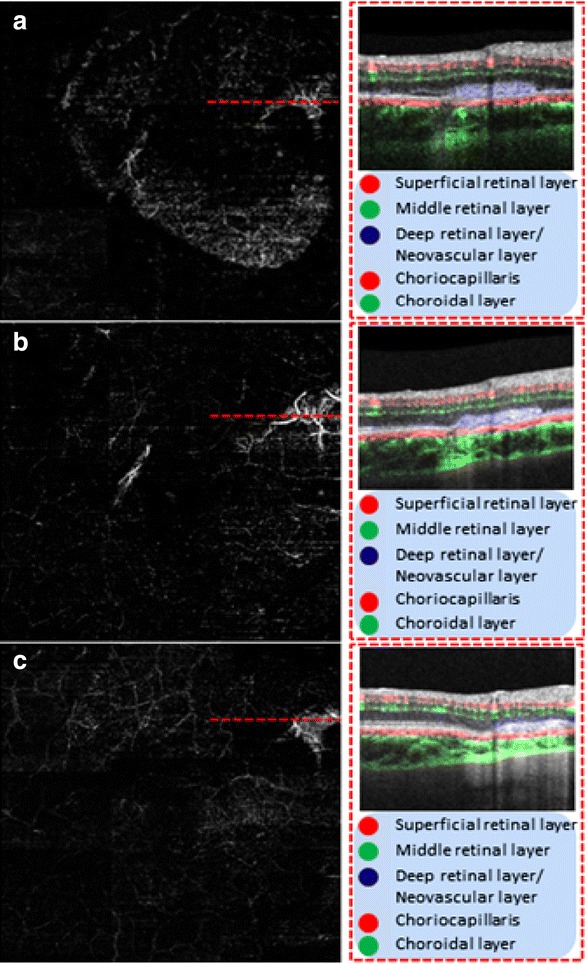
illustrates the cross sectional OCT at the level of the choroidal rupture with concern for CNVM. Shown on the left are the corresponding enface OMAG vascular maps resulted from projecting the flow signals between RPE and Bruch’s membrane, showing the neo-vessels near the rupture sites. The blood flow is color coded (representing depth) that overlays onto retinal structures. **a** Illustrated new NV flow that is represented in *blue*. **b** The NV flow diminishes in size and in **c** is faint residual flow through the regressed vessels

Our patient’s visual acuity continues to improve as the submacular hemorrhage is gradually being absorbed. On postoperative month 4, as seen on the SDOCT (Fig. 2) and OMAG (Figs. 1, 3), there was evidence of break in Bruch’s membrane however without intraretinal or subretinal blood or fluid. Patient’s last report BCVA was 20/50 in the right eye without further intervention.

## Discussion

In this study, we used OMAG technology to investigate the retinal and choroidal microvasculature in patient with CNV after a choroidal rupture. OMAG was initially used in the patient to evaluate and describe the vascular architecture of the area of traumatic choroidal rupture and breaks in Bruch’s membrane. Irregular pathology was found consistent with CNV at one of three rupture sites. We continued to follow the patient with this modality and monitor the progression and regression (probably due to resolution of the acute traumatic phase) of CNV without any intervention.

With a novel and more sensitive imaging technology associated with SD-OCT instruments, we have utilized OMAG imaging as a non-invasive modality to visualize the retinal and choroidal microvasculature without the use of intravenous injected dyes. OMAG reconstructs a three dimensional microvasculature of the retina and choroid in vivo, which allows one to analyze the dynamic behavior of a moving blood cell in a patent vessel to the level of a capillary. The imaging system is safe and noninvasive, and is capable of objectively providing detailed images with a short capture time, enabling the analysis of correlation between fundus examination and OCT angiograms, i.e., OMAG. 3D imaging for depth resolution and the ability to separate the layers as needed for the evaluation of individual vascular layers demonstrates the ability of OCT angiography to image CNV and its progression in the patient.

The OMAG may be useful as a novel screening method and early diagnostic tool for CNV in patients with choroidal rupture. This image modality may be used to evaluate changes in structure with or without intervention. By using this ability to extract and visualize retina and choroidal vasculature in CNV, OMAG imaging may help facilitate the early diagnosis of disease and possibly provide a better understanding of disease progression and efficacy of future treatment.

## Conclusion

OMAG is a non-invasive imaging modality capable of acquiring 3 dimensional retinal and choroidal microvascular maps without the use of exogenous dye. It also provides information about vascular flow of CNVM. CNVM is one of the common complications of choroidal rupture. OMAG can be used to detect and follow the progression of the CNVM. Further studies are needed to assess the role of quantitative and qualitative OCT microangiography in the evaluation and treatment of choroidal rupture.
